# Selection of CT variables and prognostic models for outcome prediction in patients with traumatic brain injury

**DOI:** 10.1186/s13049-021-00901-6

**Published:** 2021-07-17

**Authors:** Djino Khaki, Virpi Hietanen, Alba Corell, Helena Odenstedt Hergès, Johan Ljungqvist

**Affiliations:** 1 Department of Neurosurgery, Sahlgrenska University Hospital, SE-413 45 Gothenburg, Sweden; 2 Department of Clinical Neuroscience, Institute of Neuroscience and Physiology, The Sahlgrenska Academy at the University of Gothenburg, Gothenburg, Sweden; 3 Department of Anesthesia and Intensive Care, Sahlgrenska University Hospital, Gothenburg, Sweden; 4 Institute of Anesthesiology and Intensive Care Medicine, The Sahlgrenska Academy at the University of Gothenburg, Gothenburg, Sweden

**Keywords:** Traumatic brain injury, IMPACT score, CRASH score, Stockholm CT score, Marshall classification, Rotterdam scoring system, Helsinki CT score

## Abstract

**Background:**

Traumatic brain injuries (TBI) are associated with high risk of morbidity and mortality. Early outcome prediction in patients with TBI require reliable data input and stable prognostic models. The aim of this investigation was to analyze different CT classification systems and prognostic calculators in a representative population of TBI-patients, with known outcomes, in a neurointensive care unit (NICU), to identify the most suitable CT scoring system for continued research.

**Materials and methods:**

We retrospectively included 158 consecutive patients with TBI admitted to the NICU at a level 1 trauma center in Sweden from 2012 to 2016. Baseline data on admission was recorded, CT scans were reviewed, and patient outcome one year after trauma was assessed according to Glasgow Outcome Scale (GOS). The Marshall classification, Rotterdam scoring system, Helsinki CT score and Stockholm CT score were tested, in addition to the IMPACT and CRASH prognostic calculators. The results were then compared with the actual outcomes.

**Results:**

Glasgow Coma Scale score on admission was 3–8 in 38%, 9–13 in 27.2%, and 14–15 in 34.8% of the patients. GOS after one year showed good recovery in 15.8%, moderate disability in 27.2%, severe disability in 24.7%, vegetative state in 1.3% and death in 29.7%. When adding the variables from the IMPACT base model to the CT scoring systems, the Stockholm CT score yielded the strongest relationship to actual outcome. The results from the prognostic calculators IMPACT and CRASH were divided into two subgroups of mortality (percentages); ≤50% (favorable outcome) and > 50% (unfavorable outcome). This yielded favorable IMPACT and CRASH scores in 54.4 and 38.0% respectively.

**Conclusion:**

The Stockholm CT score and the Helsinki score yielded the closest relationship between the models and the actual outcomes in this consecutive patient series, representative of a NICU TBI-population. Furthermore, the Stockholm CT score yielded the strongest overall relationship when adding variables from the IMPACT base model and would be our method of choice for continued research when using any of the current available CT score models.

**Supplementary Information:**

The online version contains supplementary material available at 10.1186/s13049-021-00901-6.

## Background

Traumatic brain injury (TBI) is a major cause of death and disability worldwide [1–3]. Risk stratification and early outcome prediction is important for triage and clinical decision making as well as for communication with the patients’ relatives and for clinical audit purposes. The need for accurate outcome prediction has opened the field of prognostic modeling in TBI. Historically, prognostication relied merely on clinical information, and although this is still important, more complex models also include information about radiological features, laboratory results and biomarkers [4, 5]. The most commonly cited models, CRASH score [6] and IMPACT score [7], combine clinical and radiological information to make individual prognoses.

Studies of these models have identified four important prognostic variables following TBI: pupillary response, Glasgow Coma Scale (GCS) score, patient age, and radiological findings on computed tomography (CT) [7]. These prognostic features have been displayed graphically, enabling risk stratification in relation to outcome, and ultimately, provide a tool to aid in clinical decision making [8, 9]. The CRASH and IMPACT models are continuously validated on new patient series [10–14].

The CRASH trial was an international multicenter study which aimed to determine the effects on death and disability of a short-term corticosteroid infusion following significant head injury, and the first results were published in 2004. However, this trial eventually led to the presentation of a prognostic calculator in TBI [6]. The International Mission on Prognosis and Analysis of randomized Controlled Trials in TBI (IMPACT) studies were initiated in 2003 and continued over a 10-year period. A collaboration that commenced with the Medical Research Council of Great Britain (MRC), CRASH, led to the presentation of an enhanced prognostic calculator that included predictors of outcome in three prognostic models of increasing complexity; a Core model based on injury severity and demographics (including age, motor score and pupillary reactivity), an Extended model additionally including CT information (based on Marshall CT classification) and secondary insults (hypoxia and hypotension); and a Lab model additionally including glucose and hemoglobin values [15]. The results of CRASH and IMPACT prognostic calculators are presented as the predicted probability in percent of 6 month mortality and unfavourable outcome, where 0% responds to no risk of mortality or unfavourable outcome and 100% an extremely high risk of mortality and unfavourable outcome.

However, several other investigations have aimed to improve the classification of the CT variables, and for this study, four other CT classification scores were included in addition to the CRASH score and the IMPACT score. These scores were the Marshall CT classification [16], the Rotterdam scoring system [17], the Helsinki CT score [18] and the Stockholm CT score [19] (see Supplementary Table 1 for an overview). While the Marshall CT classification has been considered “gold standard”, albeit limitations, the Rotterdam scoring system was based on Marshall CT classification with the addition of other CT variables for a better estimated prognosis [17]. The Helsinki CT score and Stockholm CT score are similar to the previous mentioned scores, with their own individual variables [18, 19].

The aim of this investigation was to test and analyze different CT classification systems and prognostic calculators in a representative population of TBI-patients, with known outcomes, in a neurointensive care unit (NICU), to identify the most suitable CT scoring system for clinical use and continued research.

## Methods

### Patient cohort

**Fig. 1:**
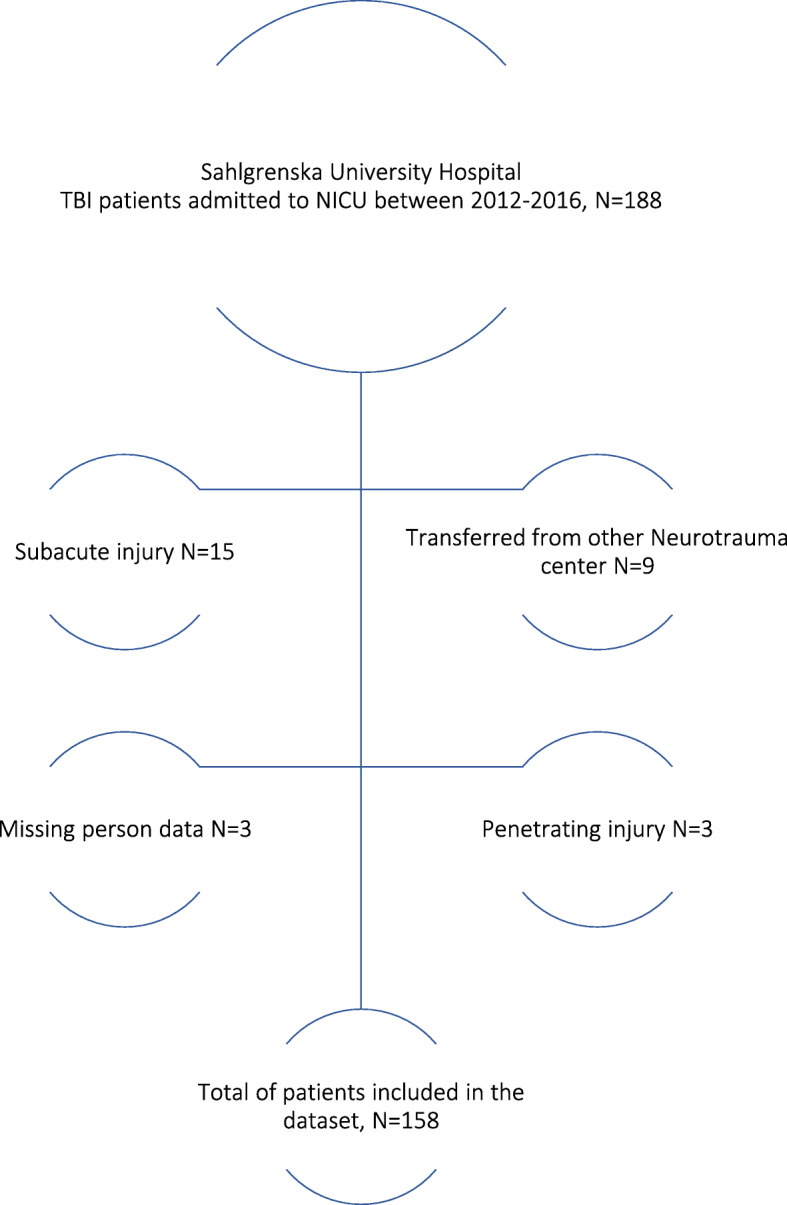
Flow-chart of inclusion and exclusion of patients

All consecutive adult patients with TBI, admitted to the neuro-intensive care unit (NICU) at the level 1 trauma center at Sahlgrenska University Hospital, in Gothenburg, Sweden from 2012 to 2016 were identified (*n* = 188), and 158 patients were included. Exclusion criteria were subacute injury, missing person data, patient transferred from another neurotrauma center and penetrating injury, see Fig. 1. Baseline data and clinical variables were retrieved from the medical records. These variables included: date of trauma, gender, age, trauma mechanism, GCS score, pupillary response, best motor response, signs of hypoxia or hypotension, blood glucose levels, hemoglobin levels (Hb), any signs of extracranial injuries, and whether or not the patient had any anticoagulatory medications.

The trauma mechanisms were classified according to the categories fall from height, assault, unclear reason of trauma (where the patient was found unconscious), and traffic accidents. Any extracranial injuries which by themselves required hospital care (in addition to the intracranial injuries) were also registered in the data material. These could include injuries to the upper and lower limbs, vertebral column, thoracic or abdominal injuries as well as facial injuries.

The traffic accidents included any accidents where a vehicle (car, bus, tram, motorcycle, bicycle, or trailer) either collided with another vehicle, with a person, or as a single vehicle accident.

Patients initially admitted to local hospitals in the area who were in need of neurointensive care were transferred to the level 1 trauma center at Sahlgrenska University Hospital and the NICU. After treatment, the patients were again transferred back to their local hospital for continued care and rehabilitation.

### Radiological CT scores and prognostic models

CT examinations were reviewed, and data for the variables of the different CT scores was registered. The first CT scans upon arrival were retrieved rather than the “worst” CT scans. The Marshall CT classification, Rotterdam scoring system, Helsinki and Stockholm CT scores were tested, and the IMPACT and CRASH prognostic calculators were applied [7].

The results of the CRASH and IMPACT calculators are presented as continuous variables from 0 to 100%, where 0% represents no risk for mortality or unfavorable outcome at 6 months and 100% a guaranteed risk for mortality or unfavorable outcome at 6 months. For this study, we dichotomized the predicted outcomes, i.e. ≤50% was assessed as favorable outcome (GOS 4–5), and > 50% was assessed as unfavorable outcome GOS [1–3].

### Outcome

Glasgow Outcome Scale (GOS) was used for global evaluation of outcome. GOS is a grading scale ranging from 1 (dead) to 5 (full recovery). An unfavorable outcome was defined as GOS 1–3 and favorable outcome as GOS 4–5. The assessment of GOS scores was based on interviews with the patients or their next of kin by a specially trained intensive care nurse (VH) one year post injury [20].

### Statistics

The statistical analyses were made using statistical software SPSS, version 25. Statistical significance was set at *p* < 0.05. The categorical variables were analyzed by frequencies and cross tables which are presented in numbers and percentages. The continuous variables are presented as medians and interquartile ranges due to non-normal distributed data. To show the correlation between the CT scoring systems and GOS, mean plots were used. The plots were made by variance analyses (one-way ANOVA). For validation and comparison of the different CT scoring systems, we used two statistical methods; logistical regression analyses and area under the receiver operating characteristic curve (AUC).

Logistical regression analyses were used to measure the association and accuracy between the different CT scoring systems and GOS. This yielded Nagelkerke’s pseudo-R2 which in turn gave a value between 0 and 1, where 0 equals no explanatory variation between the outcome and the model, and 1 equals fully explanatory variation.

Furthermore, AUC was used to compare with the measures of Nagelkerke’s pseudo-R2 which also gives a value of the variance between the outcome and the model. AUC gave a value between 0.5–1, where 0.5 indicates nothing more than random chance and 1 indicates a perfect association. To be able to perform the aforementioned analyses, GOS score was dichotomized in two ways. First GOS 1–3 versus 4–5 (unfavorable versus favorable outcome) and GOS 1 versus GOS 2–5 (dead versus alive).

Finally, a multivariable analysis by logistic regression was performed to further indicate the individual CT scoring systems’ value when added to the IMPACT base model. These variables included pupillary response, glucose, Hb level, age, and GCS on admission.

### Ethics

This study has been approved by the Swedish Ethical Review Authority (2019–04330). Formal consent from the patients was waived by the Review Authority.

## Results

158 patients with TBI were included during the study period, see Fig. 1. The mean age was 59 years and 70% were male. Fall from heights and traffic accidents were the most common trauma mechanisms (55 and 22%, respectively). The demographics, CT variables and outcomes are presented in Table 1.

**Table 1:** Patient demographics and baseline characteristics

Variable, n (%)	All patients (*n* = 158)
**Age (y), mean (SD)**	59 (43–69)
**Sex, n (%)**	
Female	47 (30)
Male	111 (70)
**PRE-ADMISSION**	
**Trauma mechanism, n** (%)	
Fall from height	87 (55)
Traffic accident	35 (22)
Assault	13 (8)
Unclear (found unconscious)	23 (15)
Extracranial injury	26 (16)
**ADMISSION**	
**Baseline data** (%)	
GCS 3–8	60 (38)
GCS 9–13	43 (27)
GCS 14–15	55 (35)
**Pupillary response** (%)	
Bilateral response	116 (73)
Unilateral response	22 (14)
No response	20 (13)
Hemoglobin (g/l), mean (SD)	138 (124–148)
Glucose (millimoles/l), mean (SD)	8 (7–10)
**Marshall CT classification** (%)	
I	0 (0)
II	33 (21)
III	34 (22)
IV	2 (1)
V + VI	89 (56)
**Rotterdam scoring system** (%)	
1	4 (2)
2	11 (7)
3	41 (26)
4	57 (36)
5	33 (21)
6	12 (8)
**Stockholm CT score, median (IQR)**	0,29 (0,16 – 0,40)
**Helsinki CT score, median (IQR)**	−0,04 (− 0,36 – 0,60)
**Stay at the Neurointensive Care Unit (days), n (median)**	8 (3–14)
**FOLLOW-UP**	
GOS*, one year after trauma (%)	
1 (Death)	47 (30)
2 (Vegetative state)	2 (1)
3 (Severe disability)	39 (25)
4 (Moderate disability)	43 (27)
5 (Good recovery)	25 (16)
Missing	2 (1)
1–3 (Unfavorable outcome)	88 (56)
4–5 (Favorable outcome)	68 (43)

*CT* Computed tomography. *GOS* Glasgow Outcome Scale. The data is presented by number (percentage) or median (interquartile range)

GCS scores were assessed upon admission, and the patients were divided into three groups depending on total score; severe TBI (GCS 3–8), moderate TBI (GCS 9–13) and mild TBI (GCS 14–15). In total, 60 patients presented with a severe TBI (38%), 43 patients with moderate TBI (27%) and 55 patients (35%) with mild TBI. Acute subdural hematomas were the most predominant finding present in 85% of the patients. The results from the prognostic calculators IMPACT and CRASH yielded a favorable IMPACT score in 54.4% of the cohort, and a favorable CRASH score in 38.0%. GOS at one year after discharge showed good recovery in 15.8%, moderate disability in 27.2%, severe disability in 24.7%, and death in 29.7%. See Table 1.

**Fig. 2:**
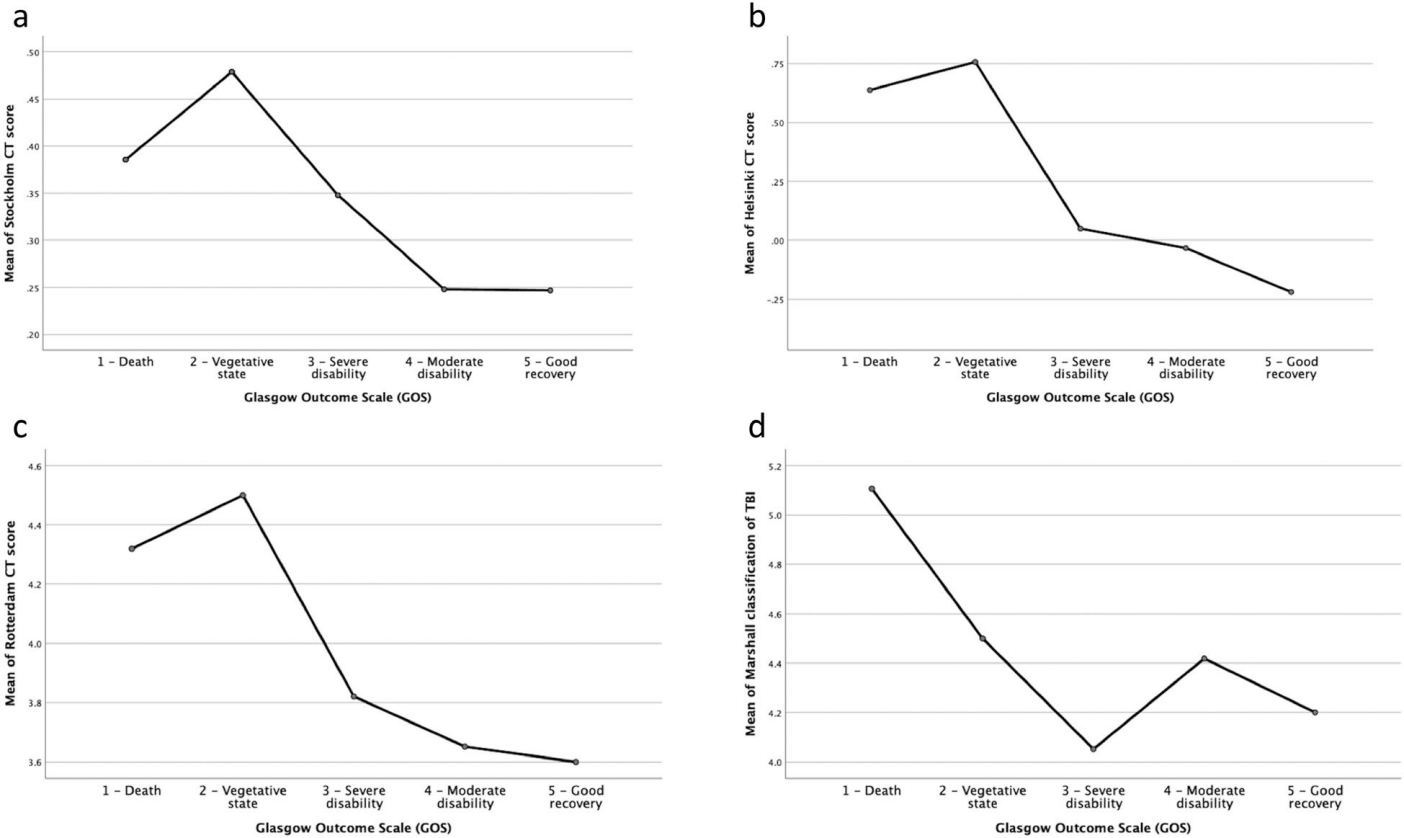
**a-d.** The relationships between the CT scores, prognostic models and outcome according to GOS

**Fig. 3:**
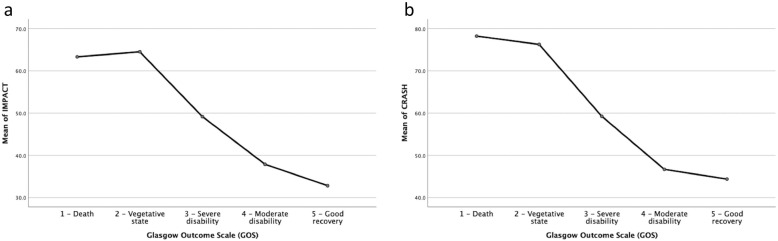
**a-b.** To illustrate the relationship between IMPACT and CRASH with outcome (GOS), means plots were used where the y-axis shows the percentage of unfavorable outcome and the x-axis shows GOS

All CT scores, as well as the IMPACT and CRASH prognostic models, were tested in relation to the actual outcomes of the patients. The relationships between CT scores, prognostic models and outcome, according to GOS, are given in Fig. 2A-D. To illustrate the relationship between the different CT scoring systems, i.e., Stockholm (A), Helsinki (B), Rotterdam (C), Marshall (D), with outcome (GOS), means plots were used where the y-axis shows the CT scoring system, and the x-axis shows GOS. This illustrates that the higher the CT scoring value, the worse the outcome and vice versa. The relationships between GOS and IMPACT and CRASH, respectively, are illustrated in Fig. 3A-B.

The statistical association and accuracy between the different CT-scoring systems and GOS are given in Table 2, for favorable versus unfavorable outcome, and in Table 3 for dead versus alive. As seen in Table 2, the Stockholm CT score remained strongest when comparing with other scoring systems, followed by the Helsinki CT score. The Helsinki score was superior in predicting survival (Table 3). When adding variables from IMPACT base model to the CT scores, the Stockholm CT score yielded statistical significance in relationship to actual outcome (see Table 4).

**Table 2:** The association and accuracy between the different CT scoring systems and GOS 1–3 (unfavourable outcome) versus GOS 4–5 (favorable outcome)

Prognostic CT scoring system	Pseudo-R^2^	AUC (95% CI)
Marshall CT classification	0,01	0,54 (0,45 – 0,63)
Rotterdam scoring system	0,06	0,61 (0,53 – 0,70)
Helsinki CT score	0,10	0,63 (0,54 – 0,71)
**Stockholm CT score**	**0,15**	**0,70 (0,61 – 0,78)**

**Table 3:** The association and accuracy between the different CT scoring systems and GOS 1 (dead) versus GOS alive [2–5]

Prognostic CT scoring system	Pseudo-R^2^	AUC (95% CI)
Marshall CT classification	0,08	0,63 (0,54 – 0,72)
Rotterdam scoring system	0,09	0,65 (0,56 – 0,74)
**Helsinki CT score**	**0,16**	**0,70 (0,61 – 0,79)**
Stockholm CT score	0,08	0,67 (0,59 – 0,76)

**Table 4:** Base model and CT scores in comparison to GOS 1–3 versus GOS 4–5

Models	Pseudo-R^2^	*P*-value (significant < 0,05)
IMPACT Base	0,330	
IMPACT Base + Marshall CT classification	0,339	0,233
IMPACT Base + Rotterdam scoring system	0,338	0,253
IMPACT Base + Helsinki CT score	0,347	0,109
**IMPACT Base + Stockholm CT score**	**0,368**	**0,020**

## Discussion

In this study, we aimed to investigate suitable CT variables and prognostic models to use in clinical practice and research in patients with known outcomes after TBI. There was a significant correlation between the predicted outcome from the scoring systems and the known outcome in the patient series. The Helsinki CT score was most accurate in predicting survival, and the Stockholm CT score was most accurate in predicting unfavorable versus favorable outcome. Furthermore, when the Stockholm CT score was added to the IMPACT base model, this yielded the strongest prognostic relationship compared to any of the other CT scoring systems.

The superior accuracy in the Stockholm and Helsinki CT scores, compared with the Marshall classification and Rotterdam scoring system, might be explained by the inclusion of additional variables, but there were also differences in how the variables were registered and how they affected prognosis. When comparing the Stockholm CT score to the other scores, there was a clear difference in the analyses of traumatic subarachnoid hemorrhage (tSAH). In the Stockholm CT score, absence or presence of intraventricular hemorrhage (IVH) analyzed, as well as the absence or presence of tSAH, both in the convexities and in the basal cisterns, in addition to the volume of the hematoma. The Marshall classification neither included tSAH nor IVH and the Rotterdam score only analyzed the absence or presence of IVH or tSAH, but did not discriminate between the two, and the Helsinki CT score analyzed only the absence or presence of IVH. Considering these factors, it is reasonable to believe that tSAH plays an important role in the prognosis of TBI patients, i.e. a worse outcome in the presence of tSAH, which has also been shown in previous studies [21]. One reason for this could be more complicated management as traumatic subarachnoid blood might case harmful complications such as vasospasm, ischemia, hydrocephalus, and electrolyte disturbances [22].

Another difference between the scoring systems concerned epidural hematomas (EDH), where the Helsinki CT score and the Stockholm CT score considered the presence of EDH a positive prognostic factor, and if present, subtracted points for EDH from the total sum; hence, yielding a more favorable outcome [19]. However, in the Rotterdam scoring system, the presence of EDH was considered a negative sign and increased the risk of poor outcome [17]. In the Marshall classification, hematomas or contusions greater than 25 cm^3^ were considered, but EDH was not specifically analyzed. Furthermore, the Stockholm CT score was the only modality which analyzed midline shift as a continuous variable and was also the only scoring system considering if signs of diffuse axonal injury (DAI) were present on CT. Even though DAI is primarily diagnosed on magnetic resonance imaging (MRI) scans, signs of DAI on CT scans could indicate severe consequences [23, 24]. Measuring midline shift as a continuous variable and including signs of DAI might explain why the Stockholm CT score was marginally more accurate than the Helsinki CT score in outcome prediction.

We found that adding relevant CT variables to the IMPACT base model yielded improved prognostic measurements. This motivates further investigations to optimize CT findings to increase prognostic accuracy which, in turn, can aid decision making in the clinical setting. From the results of this investigation, we would suggest the use of the Stockholm CT score or Helsinki CT score as the method of choice when predicting prognosis in patients with TBI. For future perspectives, we expect to see additional and more complex prognostic models in TBI, applying the results from large multi-center investigations such as the CENTER-TBI study that includes CT variables as well as biomarkers and clinical baseline data [25].

### Future prospectives in TBI prognostication

Native CT scan is part of the routine trauma protocol used at patient admission. The prognostic scores investigated in this study use findings on a native CT scan for prognostication. However, data suggests that perfusion CT scans could be superior in prognostication after TBI [26]. As CT perfusion measures cerebral vascular autoregulation, it could be useful in the guidance of TBI treatment [27, 28]. Further studies on TBI prognostication should include CT perfusion and aim to address physiology as well as associated injuries to improve prognosis and overall care for these patients.

### Strengths and limitations

The patients in this investigation were highly representative of TBI-patients in NICU. Only a small number of patients were lost to follow-up, and the standards of care adhere to the Brain trauma foundation guidelines [29] and are also routine practice in other Scandinavian and international level 1 trauma centers and NICU. Data collection was retrieved by a limited number of individuals in the team, and the radiological examinations were performed according to a routine trauma protocol and available for assessment.

One limitation was that the study was retrospective and relied on data from medical records. Moreover, when commencing this study, one aim was to compare the known patient outcome with the predicted outcome from the different prognostic models for each individual. The reason for this was to assess whether the prognostic tools yielded a poorer outcome compared to reality – or in fact a more positive outcome – and in turn, this would be another guide in the clinical setting. However, we could not find a statistical model to analyze this, due to the differences in the nature of the values of GOS and the prognostic tools. Furthermore, we assessed GOS one-year post-injury, and the prognostic models generally present outcomes at 6 months. This was a limitation in the view of comparison, and we suspect that the outcomes in our patients would be better, given the six additional months of recovery. However, the improvement beyond 6 months seems limited in reported literature [30, 31].

A possible limitation of the study was that different CT machines and protocols were used during the study period. However, for native CT scans, this has less effect compared to more advanced modalities. Additionally, a Swedish survey from 2016 showed that homogenous examination protocols for CT head scans were used in trauma and 98% used native CT scans without contrast [32]. All scanners at the different hospitals in the region underwent regular maintenance by the vendors and sequences were optimized by the hospitals for clinical evaluation of traumatic brain injuries.

## Conclusion

There was a significant correlation between the predicted outcomes from the scoring systems and the known outcomes in this patient material, representative for a NICU TBI-population. The Stockholm CT score and the Helsinki score yielded the closest relationship between the models and the actual outcomes. Furthermore, the Stockholm CT score yielded the strongest overall relationship when adding variables from IMPACT base model and would be our method of choice for continued research when using any of the current available CT scoring models.

## Supplementary Information

**Additional file 1: Supplementary Table 1.** Overview of the different prognostic calculators and CT scores.

## Data Availability

The datasets used and analyzed during the current study are available from the corresponding author on reasonable request.

## Abbreviations

CT: Computed tomography
DAI: Diffuse axonal injury
EDH: Epidural hematoma
GCS: Glasgow coma scale
GOS: Glasgow outcome scale
IVH: Intraventricular hemorrhage
MRI: Magnetic Resonance Imaging
NICU: Neurointensive Care Unit
SAH: Subarachnoid hemorrhage
SDH: Subdural hematoma
TBI: Traumatic brain injury
tSAH: Traumatic subarachnoid hemorrhage
