# Supplementary material for: Selection of CT variables and prognostic models for outcome prediction in patients with traumatic brain injury
Source: Scand J Trauma Resusc Emerg Med. 2021 Jul 17;29:94. doi: 10.1186/s13049-021-00901-6 (PMC8285829; doi:10.1186/s13049-021-00901-6)
Supplement: Supplementary file 1 — Additional file 1: Supplementary Table 1. Overview of the different prognostic calculators and CT scores. [file 13049_2021_901_MOESM1_ESM.docx]

**Supplementary material**

| **Prognostic calculator/score** | **Type of data used** | **Variables used** | | | **Prediction of outcome** |
| --- | --- | --- | --- | --- | --- |
| **IMPACT** | - Clinical data  - CT parameters  - Lab results | **Clinical data:** Age; Motor score; Pupil reaction; Hypoxia; Hypotension | **CT parameters:** CT classification by the Marshall model; tSAH; EDH  **Lab results:** Glucose; Hb | **Link to online calculator:** [IMPACT calculator](http://www.tbi-impact.org/?p=impact/calc#calcresults) | - Risk of mortality at 6 months  - Risk of unfavourable outcome at 6 months |
| **CRASH** | - Clinical data  - CT parameters | **Clinical data:** Country; Age; GCS; Pupil reaction; Major extra-cranial injury | **CT parameters:** Presence of petechial hemorrhages; Obliteration of the third ventricle or basal cisterns; SAH; Midline shift; Non-evacuated hematoma | **Link to online calculator:** [CRASH calculator](http://www.trialscoordinatingcentre.lshtm.ac.uk/Risk%20calculator/index.html) | - Risk of 14-day mortality  - Risk of unfavourable outcome at 6 months |
| **Marshall CT classification** | - CT parameters | **Diffuse injury I**  - No visible intracranial pathology  **Diffuse injury II**  - midline shift of 0 to 5 mm  - Basal cisterns remain visible  - No high or mixed density lesions >25 cm^3^ | **Diffuse injury III**  - midline shift of 0 to 5 mm  - basal cisterns compressed or completely effaced  - no high or mixed density lesions >25 cm^3^  **Diffuse injury IV**  - midline shift >5 mm  - no high or mixed density lesions >25 cm^3^ | **Evacuated mass lesion**  - any lesion evacuated surgically  **Non-evacuated mass lesion VI**  - any high or mixed density lesions >25 cm^3^ not surgically evacuated | - Descriptive rather than predictive |
| **Rotterdam scoring system** | - CT parameters | **Basal cisterns**  - Normal = 0  - compressed = 1  - absent = 2  **Midline shift**  ≤ 5 mm = 0  > 5 mm = 1 | **Epidural mass lesion**  - present = 0  - absent = 1  **Intraventricular blood or traumatic SAH**  - absent = 0  - present = 1 | **Final score** = summary of all scores + 1  - Final score 1: 0%  - Final score 2: 7%  - Final score 3: 16%  - Final score 4: 26%  - Final score 5: 53%  - Final score 6: 61% | - Risk of mortality at 6 months |
| **Helsinki CT score** | - CT parameters | **Mass lesion type(s)**  - SDH = 2  - Intracerebral hemorrhage = 2  - EDH = -3  **Mass lesion size**  - Hematoma volume   > 25 cm^3^ = 2 | **IVH**  **-** Present **=** 3  **Suprasellar cisterns**  - normal = 0  - compressed = 1  - obliterated = 5  **Sum score = -3 to 14** | **Probability:**  1/(1 + e^-LP^)  LP_mortality_= -2,666 + (0,287 x Sum score)  LP_unfavorable outcome_ = -1,636 + (0,319 x Sum score) | - Risk of mortality at 6 months  - Risk of unfavourable outcome at 6 months |
| **Stockholm CT score** | - CT parameters | **SAH in convexities**  - absent = 0  - ≤ 5mm = 1  - > 5mm = 2  **SAH in basal cisterns**  - absent = 0  - ≤ 5mm = 1  - > 5mm = 2 | **IVH**  - absent = 0  - present = 1  **SAH/IVH-score** = summed scores of subarachnoid/intraventricular hemorrhage (convexities (0–2) + basal cisterns (0–2) + intraventricular (0–1) * 2). | **Tally** = midline-shift (mm)/10 + SAH/IVH-score/2 − 1 (if EDH) +1 (if DAI) + 1 (if dual-sided SDH) + 1  **Probability score for unfavorable outcome = 1/(1 + e^(3.5-1.1*tally)^)** | - Best outcome at 1 year (16) |

Supplementary table 1. Overview of the different prognostic calculators and CT scores.
